# Hyperthermia Prevents *In Vitro* and *In Vivo* Biofilm Formation on Endotracheal Tubes

**DOI:** 10.1128/spectrum.02807-22

**Published:** 2022-12-06

**Authors:** Marta Palau, Estela Muñoz, Nieves Larrosa, Xavier Gomis, Ester Márquez, Oscar Len, Benito Almirante, Joan Gavaldà

**Affiliations:** a Antibiotic Resistance Laboratory, Vall d’Hebron Research Institute, Infectious Diseases Department, Vall d’Hebron University Hospital, Barcelona, Spain; b Spanish Network for Research in Infectious Diseases (REIPI RD19/0016), Instituto de Salud Carlos III, Madrid, Spain; c CIBERINFEC, ISCIII-CIBER de Enfermedades Infecciosas, Instituto de Salud Carlos III, Madrid, Spain; d Microbiology Department, Vall d’Hebron University Hospital, Barcelona, Spain; e Infectious Diseases Department, Vall d’Hebron University Hospital, Barcelona, Spain; Weizmann Institute of Science

**Keywords:** animal model, biofilm, endotracheal tube, hyperthermia, prevention, ventilator-associated pneumonia

## Abstract

There is currently an urgent need to find new strategies to tackle antimicrobial resistance and biofilm-related infections. This study has two aims. First, we evaluated the *in vitro* efficacy of hyperthermia in preventing biofilm formation on the surfaces of polyvinyl chloride discs. Second, we assessed the *in vivo* efficacy of hyperthermia in preventing biofilm formation in endotracheal tubes (ETTs) of a rabbit model. For the *in vitro* studies, nine clinical extensively drug-resistant/multidrug-resistant Gram-negative isolates of Acinetobacter baumannii, Klebsiella pneumoniae, and Pseudomonas aeruginosa and three clinical methicillin-resistant Staphylococcus aureus strains were studied. For biofilm formation, an adhesion step of 30 or 90 min followed by a growth step of 24 h were performed with application of one, two, and three pulses at 42°C for 15 min each pulse after the adhesion step. For the *in vivo* studies, New Zealand rabbits were intubated with ETTs previously colonized with K. pneumoniae or P. aeruginosa strains, and three pulses at 42°C for 15 min were applied after the adhesion step. The application of three pulses at 42°C for 15 min each pulse was needed to achieve the prevention of the *in vitro* biofilm formation of 100% of the tested strains. The application of heat pulses in a rabbit intubation model led to biofilm prevention of 85% against two K. pneumoniae strains and 80% against two P. aeruginosa strains compared to the control group. Hyperthermia application through pulses at 42°C could be a new nonantibiotic strategy to prevent biofilm formation in ETTs.

**IMPORTANCE** Biofilm-producing microorganisms are considered medically crucial since they cause 80% of the infections that occur in the human body. Medical devices such as endotracheal tubes (ETTs) can act as a reservoir for pathogens providing the surface to which microorganisms can adhere and cause biofilm-associated infections in critically ill patients. This biofilm has been related with the development of ventilator-associated pneumonia (VAP), with an incidence of 8 to 28%, a mortality rate up to 17% and its associated high extra costs. Although some VAP-preventive measures have been reported, they have not demonstrated a significant reduction of VAP incidence. Therefore, we present a new nonantibiotic strategy based on hyperthermia application to prevent biofilm formation inside ETTs. This technology could reduce VAP incidence, intubation duration, hospital and intensive care unit (ICU) length stays, and mortality rates. Consequently, this could decrease the antibiotics administered and influence the impact of antibiotic resistance in the ICU.

## INTRODUCTION

Medical device-related infections represent almost one-fourth of nosocomial infections, which represent an important health care problem owing to their high mortality (1). Ventilator-associated pneumonia (VAP) is considered the most severe form of nosocomial pneumonia and contributes to the development of complications in patients. It has a high prevalence in mechanically ventilated patients in intensive care units (ICU), with an estimated incidence that varies between 8 and 28% (2), which increases with the duration of ventilation, and associated mortality rate of 3 to 17% (3). Endotracheal tubes (ETTs) have been reported to act as a reservoir for microbial pathogens, providing a perfect environment for the colonization and adhesion of microorganisms on the distal part of the ETT, forming a biofilm (4, 5). Biofilms are defined as an assemblage of cells enclosed by a self-produced matrix of extracellular polymeric substances (EPS; including polysaccharides, proteins, lipids, and extracellular DNA) attached to a biotic or nonbiotic surface (6, 7). Biofilms can develop rapidly after intubation, forming detectable antibiotic-tolerant structures within 24 h, or they can be dislodged and travel down to the lung, causing critical infections (4, 8). The presence of the EPS barrier and slow growth that occurs confer tolerance to antibiotics (6). In addition to tolerance, antibiotic resistance can be enhanced in biofilms owing to the dissemination of resistance genes between bacterial cells through horizontal gene transfer (6). Moreover, some studies have reported data that correlate the formation of biofilms in the ETT with the development of VAP, since the same microorganisms causing VAP were isolated from ETT biofilms (9, 10); in fact, some authors have suggested that VAP should be renamed ETT-associated pneumonia (9–11).

In the last decade, VAP-preventive measures have been reported and applied in clinical practice, including the use of techniques to avoid respiratory infections in intubated patients (12). Although these measures have allowed better welfare, they have not been sufficient to reduce VAP incidence (13, 14). Moreover, strategies to reduce biofilm formation inside ETTs have been studied, such as ETTs coated with organic polymers, metals, composites, peptides, photocatalytic antibacterial materials, etc. (4, 8, 15). However, more evidence is required before recommending coated ETTs in clinical practice, since some studies using them have not demonstrated a significant reduction in VAP (4, 16) or a reduction of the intubation duration, hospital and ICU stays, or mortality rates (17). Therefore, new preventive or treatment strategies need to be developed to decrease the risk of ETT-related infections in critically ill patients (4).

Heat has been used as an antibacterial strategy for hundreds of years, such as for liquid food sterilization (>100°C), pasteurization of milk (>60°C), or sterilization of medical equipment surfaces by autoclaving (>120°C) (18). We define hyperthermia therapy as the treatment of a disease by increasing the temperature above the normal (between 41 and 50°C) for a certain period of time (19, 20). However, the effect of heat is always dependent on different factors such as the duration, homogeneity of the temperature, tissue type, and context of the treatment (19, 20). Some authors have reported inactivation of bacteria by heat treatment (21–25). Although most studies have focused on planktonic bacteria (21–23), there is some evidence of the antibiofilm activity of heat (24, 25).

Due to this crucial situation, since the formation of biofilms inside the ETT is considered a risk factor for the development of VAP (8), we present a new strategy to prevent the formation of biofilms inside the ETT based on the application of local heat.

## RESULTS

### *In vitro* studies.

**FIG 1:**
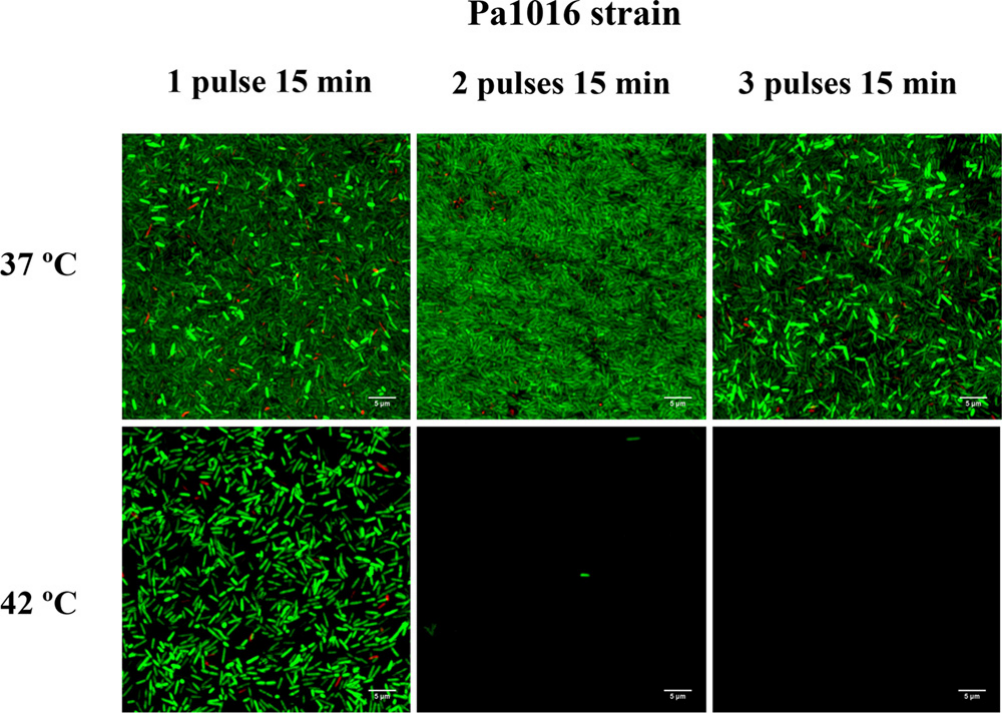
Example of the *in vitro* efficacy of hyperthermia by applying one, two, or three pulses at 42°C for 15 min each pulse after an adhesion step of 30 min against a biofilm-producing strain of P. aeruginosa (Pa1016). Images were obtained by using confocal laser scanning microscopy and a Live/Dead staining kit. Red fluorescence, dead cells; green fluorescence, live cells (visualized at ×60 magnification).

**FIG 2:**
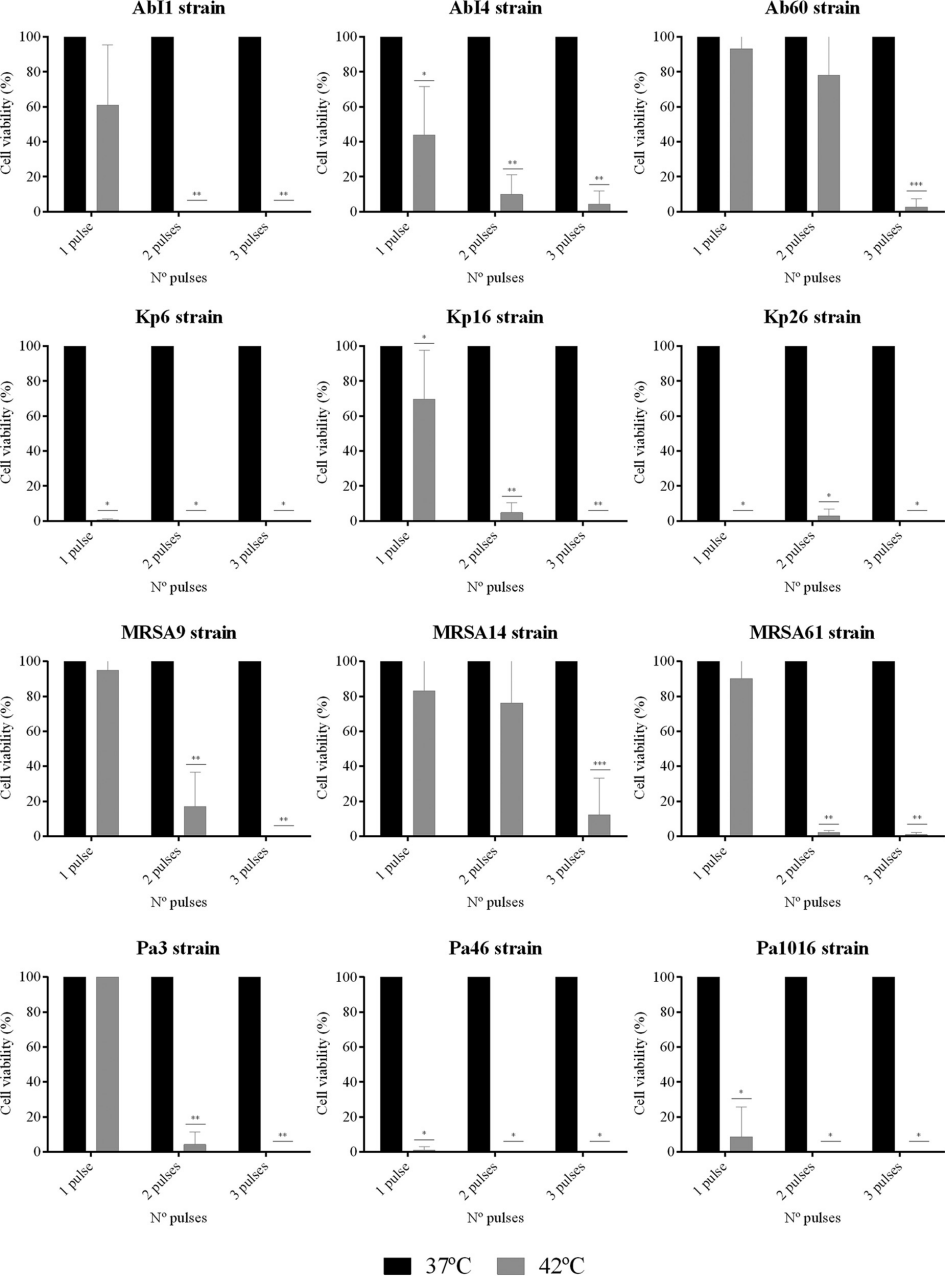
Efficacy of hyperthermia by applying one, two, or three pulses at 42°C for 15 min each pulse after an adhesion step of 30 min against different biofilm-producing strains (AbI1, AbI4, Ab60, Kp6, Kp16, Kp26, MRSA9, MRSA14, MRSA61, Pa3, Pa46, and Pa1016). Cell viability was quantified from the images obtained by using confocal laser scanning microscopy and a Live/Dead staining kit and are expressed as mean percentages ± the standard deviations. *, *P* ≤ 0.05 versus one, two, or three pulses at 37°C for 15 min; **, *P* ≤ 0.05 versus one, two, or three pulses at 37°C for 15 min and 1 pulse at 42°C for 15 min; ***, *P* ≤ 0.05 versus one, two, or three pulses at 37°C for 15 min, one pulse at 42°C for 15 min, and two pulses at 42°C for 15 min.

**FIG 3:**
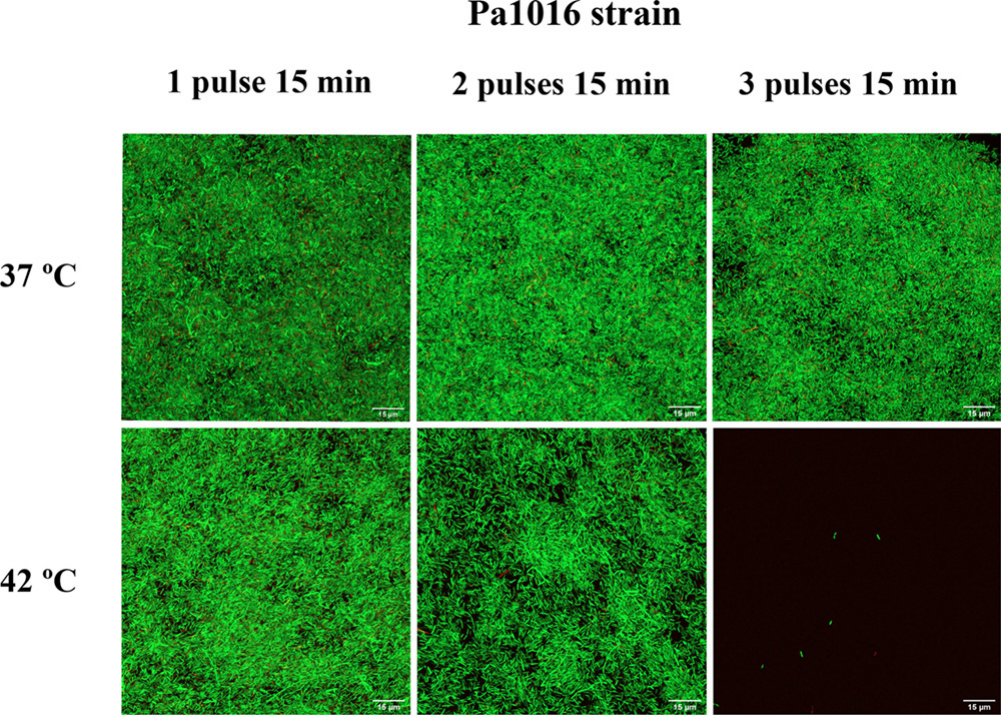
An example of the *in vitro* efficacy of hyperthermia by applying one, two, or three pulses at 42°C for 15 min each pulse after an adhesion step of 90 min against a biofilm-producing strain of P. aeruginosa (Pa1016). Images were obtained by using confocal laser scanning microscopy and a Live/Dead staining kit. Red fluorescence, dead cells; green fluorescence, live cells (visualized at ×60 magnification).

**FIG 4:**
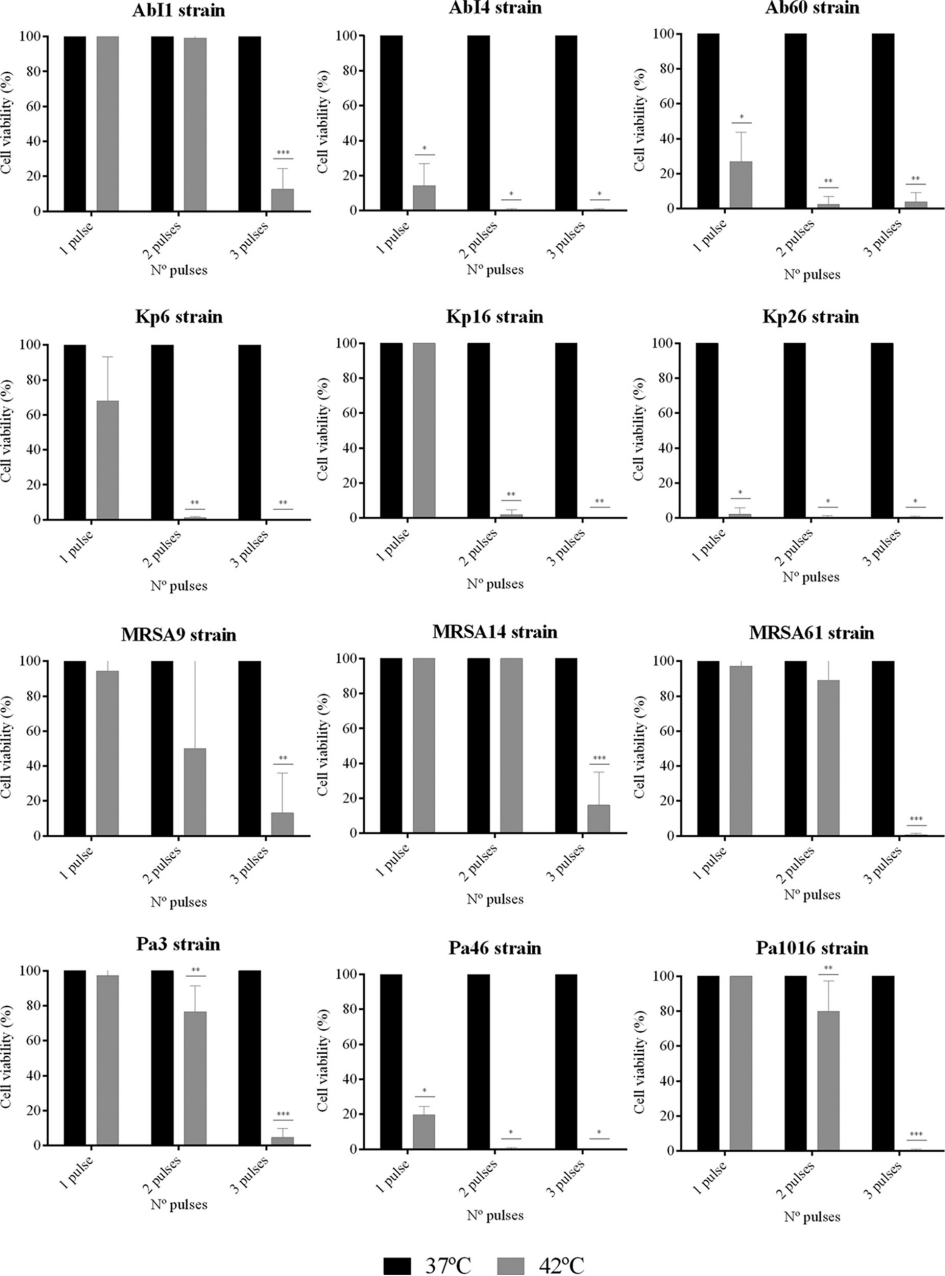
Efficacy of hyperthermia by applying one, two, or three pulses at 42°C for 15 min each pulse after an adhesion step of 90 min against different biofilm-producing strains (AbI1, AbI4, Ab60, Kp6, Kp16, Kp26, MRSA9, MRSA14, MRSA61, Pa3, Pa46, and Pa1016). Cell viability was quantified from the images obtained by using confocal laser scanning microscopy with Live/Dead staining, and results are expressed as mean percentages ± the standard deviations. *, *P* ≤ 0.05 versus one, two, or three pulses at 37°C for 15 min; **, *P* ≤ 0.05 versus one, two, or three pulses at 37°C for 15 min and one pulse at 42°C for 15 min; ***, *P* ≤ 0.05 versus one, two, or three pulses at 37°C for 15 min, one pulse at 42°C for 15 min, and two pulses at 42°C for 15 min.

The *in vitro* efficacy of hyperthermia against biofilm-producing strains is presented in Fig. 1 to 4.

As shown in Fig. 1 and 3, the application of hyperthermia to bacterial strains growing on polyvinyl chloride (PVC) discs did not affect their morphology, since the shape and size of the treated cells were not altered compared to those of the untreated group. Moreover, the Live/Dead staining results revealed the ability of hyperthermia to prevent biofilm formation on the surface of PVC discs, as after applying two or three pulses at 42°C for 15 min each pulse, only a few cells were attached in comparison with the control group. Although only one example of the tested strains is shown in Fig. 1 and 3, the same results were obtained for all other clinical strains tested.

The prevention of biofilm formation after a 30- and 90-min adhesion step was achieved by applying a maximum of three pulses at 42°C for 15 min each pulse against all the extensively drug-resistant (XDR) and multidrug-resistant (MDR) clinical strains tested (Fig. 2 and 4). Specifically, the application of one or two pulses at 42°C prevented biofilm formation by 83% of the strains, including AbI1, AbI4, Kp6, Kp16, Kp26, Pa3, Pa46, Pa1016, MRSA9, and MRSA61, after a 30 min-biofilm attachment step (Fig. 2). The number of heat pulses was increased to prevent biofilm formation after an adhesion step of 90 min, since the tested strains were less susceptible to the hyperthermia effect (Fig. 4). In this case, one or two pulses at 42°C prevented biofilm formation in half of the tested strains (AbI4, Ab60, Kp6, Kp16, Kp26, and Pa46), whereas three pulses at 42°C were needed to prevent biofilm formation in the remaining strains (AbI1, MRSA9, MRSA14, MRSA61, Pa3, and Pa1016).

### *In vivo* studies.

**FIG 5:**
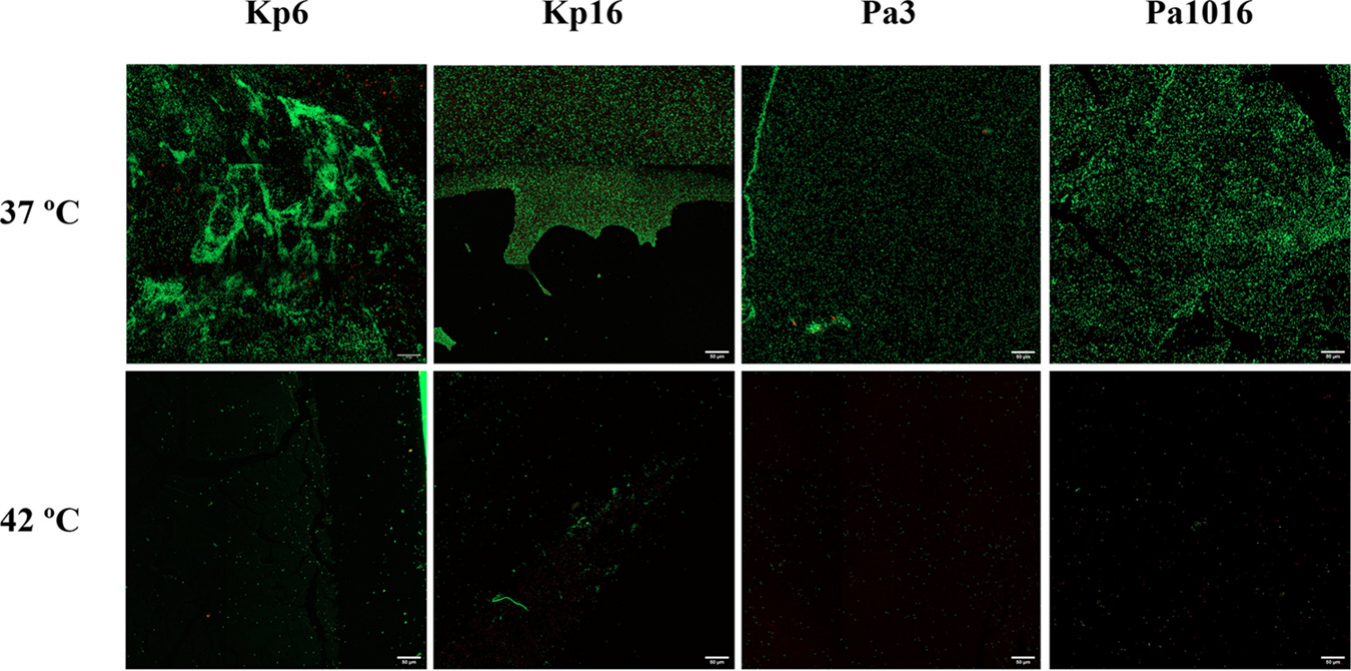
*In vivo* efficacy of hyperthermia by applying three pulses at 42°C for 15 min each pulse after an adhesion step of 30 min against two biofilm-producing strains of K. pneumoniae (Kp6 and Kp16) and P. aeruginosa (Pa3 and Pa1016) in an ETT in a rabbit intubation model. Images were obtained by confocal laser scanning microscopy and a Live/Dead staining kit. Z-stacks of multiple consecutive tiles of the sample were scanned by using the tiles scanning package of the ZEN Blue 3.3 software. A maximum projection was obtained using the same software. Red fluorescence, dead cells; green fluorescence, live cells (visualized at ×40 magnification). ETT, endotracheal tube.

**FIG 6:**
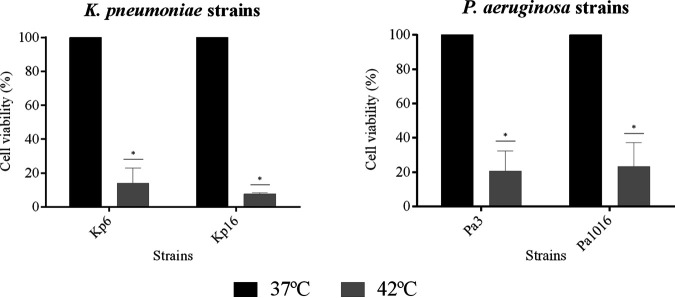
Efficacy of hyperthermia by applying three pulses at 42°C for 15 min each pulse after an adhesion step of 30 min against two biofilm-producing strains of each K. pneumoniae (Kp6 and Kp16) and P. aeruginosa (Pa3 and Pa1016). Cell viability was quantified from the images obtained by confocal laser scanning microscope using a Live/Dead staining kit, and results are expressed as mean percentages ± the standard deviations. *, *P* ≤ 0.05 versus three pulses at 37°C for 15 min.

The results obtained using the intubation rabbit model are presented in Fig. 5 and 6. As depicted, the application of hyperthermia has a powerful effect and prevents the biofilm formation in ETTs colonized with Klebsiella pneumoniae or Pseudomonas aeruginosa strains.

As shown in Fig. 5, the application of hyperthermia inside the ETT in the intubated rabbit model did not alter the shape and size of the treated bacterial cells. Moreover, the Live/Dead staining results revealed the preventive effect of hyperthermia on the formation of biofilms in the lumen of the ETT, since only a few cells were attached compared to the control group (37°C) after applying three pulses at 42°C for 15 min each pulse. Specifically, the application of hyperthermia led to a biofilm prevention of more than 85% against two K. pneumoniae strains (Kp6 and Kp16) and more than 80% against two P. aeruginosa strains (Pa3 and Pa1016) in comparison to the control group (Fig. 6).

## DISCUSSION

This study has two aims. First, we evaluated the *in vitro* efficacy of hyperthermia in preventing biofilm formation on the surface of PVC discs after an adhesion step of 30 or 90 min against a wide range of clinical strains. Next, the *in vivo* efficacy of hyperthermia for preventing biofilm formation inside ETTs was assessed in an experimental model using an intubated rabbit.

In this article, we demonstrated the *in vitro* and *in vivo* efficacy of hyperthermia for preventing biofilm formation in ETTs. Specifically, we have proven the high ability of hyperthermia to avoid the formation of biofilms of Acinetobacter baumannii, K. pneumoniae, P. aeruginosa, and methicillin-resistant Staphylococcus aureus (MRSA) strains growing on the surface of PVC, which is the material from which ETTs are made. Moreover, the powerful effect of hyperthermia to prevent the biofilm formation was also observed in an intubation rabbit model using ETTs colonized with K. pneumoniae or P. aeruginosa strains.

The effect of heat against bacterial strains growing in planktonic cultures has been studied by Tsuchido et al. (26) and Menezes et al. (27). Both studies have shown that high temperatures (55 and 46°C, respectively) can affect bacterial proliferation and mobility and consequently increase the autolysis and permeability of bacterial cells (26, 27). Moreover, another study performed by Eshraghi et al. (28) added the effect of decreasing protein synthesis when Escherichia coli strains were exposed to heat (48°C).

Focusing on our *in vitro* assays, we prevented biofilm formation of all the clinical strains tested after an adhesion step of 30 or 90 min by applying only three pulses at 42°C for 15 min each pulse against a wide range of clinical bacterial strains of A. baumannii, K. pneumoniae, P. aeruginosa, and MRSA. Our results are in agreement with those reported by Richardson et al. (29). This study showed that an elevation in temperature (45 to 60°C) caused a significant reduction in the biofilm formation by S. aureus, K. pneumoniae, and Staphylococcus epidermidis on the surfaces of catheters. The main difference between these studies and ours is that, in our case, we only needed 42°C to prevent the biofilm formation of clinical S. aureus strains obtained from patients, whereas they used higher temperatures and ATCC strains.

Moreover, similar studies were performed by O’Toole et al. (25), who observed that biofilm formation decreased when the temperature (37 to 80°C) and exposure time (1 to 30 min) were increased. Furthermore, they studied two different biofilm cultures: one using stirring plates and the other using drip flow reactors (25). As observed in previous studies (30), their results highlight the impact of the substrate used for biofilm formation on the efficacy of the antimicrobial agent. That is the reason why in our *in vitro* studies, the biofilm formation was performed using PVC as a substrate, being the material from which ETTs are made.

Furthermore, our *in vitro* studies have shown that the number of heat pulses must be increased when the adhesion step is longer than 30 min (90 min). Although the application of one or two shots at 42°C prevented the biofilm formation of almost all the strains after an adhesion step of 30 min, three shots at 42°C for 15 min each pulse were needed to prevent biofilm formation in 50% of the studied strains after an adhesion step of 90 min. Our results are in agreement with those published by Chang et al. (18), who also observed that longer exposure times (more than 30 min) resulted in low biofilm formation, whereas a mature biofilm was still observed by confocal laser scanning microscopy when a shorter exposure time (<10 min) was applied (18).

Some studies have concluded that hyperthermia, as well as fever, in addition to its antimicrobial activity, can cause activation of the immune system by stimulating the production of interferon, mobility of leukocytes, and leukocyte killing by microorganisms (31, 32). Therefore, determining the impact of heat on infections should be more realistic *in vivo* (33).

Focusing on our *in vivo* studies, hyperthermia application has a powerful effect of preventing biofilm formation in ETTs up to 80% in two strains of P. aeruginosa and two strains of K. pneumoniae. In our study, the final temperature inside the ETT was 42°C, which it is not harmful to the tissues but is effective for biofilm prevention by interfering with bacterial adhesion on the surface of ETTs. Our results correlate with those published by Storm et al. (34), in which they confirmed that the application of temperatures below 45°C were safe for the lungs (34).

In recent decades, some strategies to avoid biofilm formation in ETTs have been studied, such as antibacterial-coated ETTs, with silver-coated ETT being the most studied (16, 35). These studies have demonstrated high biofilm reduction by changing the bacterial adhesion conditions, which is in concordance with our results. Although some of these studies have achieved a higher reduction in biofilm formation, our strategy maintains the use of standard ETTs and avoids using compounds that might be released into the body and cause side effects.

Some clinical studies have found that pathogenic microorganisms responsible for the development of VAP in intubated patients are isolated from biofilms formed in ETTs which are present in tracheal secretions (5, 9). Thorarinsdottir et al. (35) highlighted that high levels of biofilm formation on ETTs are significantly associated with the development of VAP and mentioned P. aeruginosa and K. pneumoniae among the frequent species isolated from biofilms formed in ETTs (35).

Some coated-ETT preclinical studies have shown that disrupting bacterial adhesion inside the ETT and preventing biofilm formation inside it seems to lead to VAP reduction, concluding that preventing biofilm formation in ETTs might be an outstanding solution for decreasing VAP infections (9, 35). Therefore, we expect that our technology could favor VAP reduction in intubated patients, although further studies and clinical assays are needed.

Some studies have highlighted the enhanced antimicrobial activity of a combination of antibiotics and hyperthermia in biofilms (36–38). Two of the proposed mechanisms by which hyperthermia increases antibiotic efficacy are enhancing antibiotic transport through the EPS present in biofilms at high temperatures and the stimulation of bacterial metabolism by heat (36). Thus, the addition of antibiotics to hyperthermia could be a challenge for future studies.

Finally, future preclinical efficacy studies in pig and sheep models, as well as further clinical trials need to be done to determine the optimum number of heat pulses for the patient, the length duration of each pulse and the number of times the treatment will be repeated during the patient’s stay in the ICU. Moreover, more safety studies need to be addressed to determine the effect of long exposure times of hyperthermia, as well as to monitor animals over time after the hyperthermia application.

In conclusion, our study supports the idea that the application of hyperthermia at 42°C could be a new nonantibiotic strategy to prevent infections caused by XDR P. aeruginosa, XDR A. baumannii, ESBL K. pneumoniae, and MRSA strains growing in ETT biofilms. This new technology could decrease the incidence of VAP and its morbidity and mortality. Consequently, this could decrease the amount of antibiotics administered to intubated patients and influence the impact of antibiotic resistance in ICUs.

## MATERIALS AND METHODS

### Strains and resistance mechanisms.

For *in vitro* biofilm formation assays, nine clinical extensively drug-resistant (XDR) and multidrug-resistant (MDR) Gram-negative isolates were studied: three strains of Acinetobacter baumannii (AbI1, AbI4, and Ab60), three strains of Klebsiella pneumoniae (Kp6, Kp16, and Kp26), and three strains of Pseudomonas aeruginosa (Pa3, Pa46, and Pa1016). Sequence typing, main acquired β-lactam resistance mechanisms, and results of antibiotic susceptibility testing (AST) of these Gram-negative strains are summarized in Table 1. Antimicrobial susceptibility was studied using the disk diffusion test (i2a, Montpellier, France) according to the European Committee on Antimicrobial Susceptibility Testing (EUCAST) guidelines (39). In addition, susceptibility to antibiotics that are potentially active against some of these XDR isolates (ceftazidime, ceftazidime-avibactam, ceftolozane-tazobactam, piperacillin-tazobactam, cefiderocol, meropenem, meropenem-vaborbactam, imipenem/relebactam, amikacin, ciprofloxacin, trimethoprim-sulfamethoxazole, and colistin) was determined in duplicate by the microdilution technique using a Sensititre Gram-negative GN4F AST Plate (Thermo Fisher Diagnostics S.L.U., Madrid, Spain). Discordance between previous techniques was confirmed using a gradient test (Etest, bioMérieux SA, Marcy l’Etoile, France). The American Type Culture Collection (ATCC) strains P. aeruginosa 27853, Escherichia coli 25922, and K. pneumoniae 700603 and 2814 were used as quality control strains to determine the MICs. Besides, three Gram-positive clinical strains of methicillin-resistant Staphylococcus aureus (MRSA) were studied (MRSA9, MRSA14, and MRSA61).

**TABLE 1:** Characteristics of the Gram-negative isolates used in the study^*a*^

Strain	ST	Description^*b*^	Microdilution technique, MIC (mg/L)									Disk diffusion, zone diameter (mm)		
			CTZ	CTV	CTT	PIT	MER	MEV	IMR	CIP	COL	FDC	AMI	TRS
AbI1	103	NDM-2, OXA-51	>32 ND	>8/4 ND	>8/4 ND	>64/4 ND	>16 R	8/8 ND	>8/4 R	4 R	0.5 S	16.1 R	6 R	6 R
AbI4	2	OXA-51	>32 ND	>8/4 ND	>8/4 ND	>64/4 ND	>16 R	>8/8 ND	>8/4 R	>4 R	1 S	20.5 R	22 S	6 R
Ab60	38	OXA-51	2 ND	>8/4 ND	>8/4 ND	>64/4 ND	2 S	4/8 ND	0.5/4 S	1 I	0.5 S	24.8 S	S	S
Kp6	ND	IMP, CTX-M	>32 R	>8/4 R	>8/4 R	>64/4 R	8 R	8/8 R	0.5/4 S	2 R	0.125 S	25.5 S	20 S	6 R
Kp16	ND	DHA	>32 R	0.5/2 S	2/4 S	>64/4 R	0.25–0.5 S	0.05/8 S	2/4 S	0.5 I	0.5 S	23.2 S	26 S	30 S
Kp26	ND	CTX-M	>32 R	0.75/2 S	2/4 S	8/4 S	0.05 S	0.05/8 S	0.5/4 S	>4 R	0.5 S	20.4 R	23 S	6 R
Pa3	235	VIM-2	>32 R	> 8/4 R	>8/4 R	>64/4 R	>16 R	>8/8 R	>8/4 R	>4 R	2 S	24.6 S	11 R	6 R
Pa46	111	VIM-2	>32 R	>8/4 R	>8/4 R	>64/4 R	>16 R	>8/8 R	>8/4 R	>4 R	1 S	23.3 S	12 R	6 R
Pa1016	175		32 R	2/4 S	2/4 S	>64/4 R	16 R	>8/8 R	4 S	>4 R	4 S	25.6 S	24 S	6 R

a ST, sequence type; MIC, minimum inhibitory concentration; ESBL, extended-spectrum β-lactamase; CTZ, ceftazidime; CTV, ceftazidime-avibactam; CTT, ceftolozane-tazobactam; PIT, piperacillin-tazobactam; MER, meropenem; MEV, meropenem-vaborbactam; IMR, imipenem/relebactam; CIP, ciprofloxacin; COL, colistin; FDC, cefiderocol; AMI, amikacin; TRS, trimethoprim-sulfamethoxazole; ND, not determined; R, resistant; S, susceptible.

b That is, third-generation cephalosporin and carbapenemase resistance mechanisms.

For the *in vivo* efficacy assays, two strains of P. aeruginosa (Pa3 and Pa1016) and two strains of K. pneumoniae (Kp6 and Kp16) were studied. All studied strains were isolated from patients at the Vall d’Hebron University Hospital (VHUH). All strains were stored in skimmed milk at −80°C in cryovial storage containers. Prior to each experiment, the strains were plated on trypticase soy agar (bioMérieux SA, Marcy l’Etoile, France) and incubated at 37°C for 24 h.

### Substrates used for the biofilm formation.

For the *in vitro* studies, PVC discs (diameter, 15 mm; thickness, 0.5 mm; Servicio Estación S.A, Barcelona, Spain) were used as the substrates for biofilm formation. To improve biofilm formation, K. pneumoniae strains were grown on thicker PVC discs (diameter, 15 mm; thickness, 1 mm; Servicio Estación S.A).

For the *in vivo* studies, Hi-Contour oral/nasal cuffed ETTs (internal diameter, 3.5 mm; outer diameter, 4.9 mm; Covidien, Mansfield, MA) were used.

### *In vitro* studies.

**(i) Biofilm formation on PVC discs.** For biofilm formation on the surface of the discs, the protocol described by Chandra et al. (40) was followed with some modifications. Biofilm-producing strains (AbI1, AbI4, Ab60, Kp6, Kp16, Kp26, Pa3, Pa46, Pa1016, MRSA9, MRSA14, and MRSA61) were separately grown overnight in tryptic soy broth (TSB; Becton, Dickinson and Company, Le Pont de Claix, France) at 37°C and 60 rpm. After centrifuging and washing the cell suspension three times with sterile phosphate-buffered saline (PBS [pH 7.2]; Merck, Germany), an inoculum of 1 × 10^7^ CFU/mL was prepared with PBS (pH 7.2). Next, 4 mL of the inoculum and PVC discs was added to each well of a 12-well plate (Sarstedt AG & Co., Nümbrecht, Germany). The plates were incubated for 30 or 90 min at 37°C (adhesion step). Hyperthermia was applied by placing the discs on a heating plate (Sanara, Barcelona, Spain). The discs were then placed in a new plate containing 4 mL of TSB per well. Finally, the plate was incubated for 24 h at 37°C with stirring at 60 rpm (growth step).

**(ii) Application of the hyperthermia.** After the adhesion step (30 or 90 min) of biofilm formation on the surface of the PVC discs, three discs were placed in a new 12-well plate and incubated at 37°C (control group). The other three discs were placed on a heating plate, where one, two, and three pulses at 42°C for 15 min each pulse were applied. Between pulses, the discs were incubated at 37°C for 30 min. A thermometer (Omega Instruments, Manchester, United Kingdom) and thermocouple probe (Sanara) were used to verify the temperature of the control disc during the experiment. During the application of the pulses and between pulses, all the discs (treated and control) were without culture medium.

After the treatment (one, two, or three pulses), the discs were placed in a new plate containing 4 mL of TSB to follow the growth step, which consists of an incubation at 37°C for 24 h with stirring at 60 rpm. The efficacy of hyperthermia pulses was then evaluated.

**(iii) Evaluation of the hyperthermia efficacy against biofilm-producing strains.** The efficacy of hyperthermia was evaluated using a LSM980 confocal laser scanning microscope (CLSM; Zeiss, New York, NY) with excitation wavelengths of 488 and 568 nm and a magnification of ×60.

Biofilms were stained using a Live/Dead BacLight viability kit (Molecular Probes/Invitrogen, Leiden, The Netherlands) according to the manufacturer’s instructions, which consisted of staining the discs with 200 μL of a mixture of SYTO 9 (3.34 mM solution in dimethyl sulfoxide [DMSO]) and propidium iodide (20 mM solution in DMSO) dyes, followed by incubation at 25°C in the dark for 30 min. The mixture was prepared with 3 μL of each dye in 1 mL of sterile-distilled water. Three areas of the biofilm on each PVC disc were scanned with a 2-μm step size, and z-stacks of the image were defined in order to collect the information of all the layers of the biofilm. Simultaneous dual-channel imaging was used to display green (live cells) and red (dead cells) fluorescence. ZEN Blue 3.3 software was used to create a maximum projection of the formed biofilms in a single plane, and ImageJ 1.45s software package was used to calculate the value of live (green) cells. The cell viability is expressed as the percentage mean of the hyperthermia pulse group versus the control group and analyzed using one-way analysis of variance and the Tukey’s *post hoc* test. Statistical analysis was performed using the Statistical Package for the Social Sciences (SPSS, Inc., Chicago, IL). *P* values of ≤0.05 were considered statistically significant.

### *In vivo* studies.

**(i) Animals.** For *in vivo* studies, 40 New Zealand White male rabbits (Granja Cunícola San Bernardo, Navarra, Spain) weighing between 2 and 2.3 kg were housed individually in regulation cages, provided with water and food *ad libitum* throughout the duration of the experiments, under a reversed 12-h/12-h light/dark cycle.

The experimental protocol was approved by the Animal Experimentation Ethics Committee of the Vall d’Hebron Research Institute (registration number 37/20 CEEA) and the Ministry of Environment of the Catalan Government (registration number 11098).

**(ii) Colonization of the ETTs.** First, the biofilm-producing strains (Kp6, Kp16, Pa3, and Pa1016) were separately grown overnight in TSB at 37°C and 60 rpm. After the cell suspension was centrifuged and washed three times with sterile PBS (pH 7.2), an inoculum of each strain of 1 × 10^7^ CFU/mL was prepared with PBS (pH 7.2). Different ETTs were then filled and sealed with the inoculum of each strain under sterile conditions, followed by incubation for 30 min at 37°C (adhesion step). The inoculum was then emptied from the ETT.

**(iii) Anesthesia.** Animals were anesthetized by an intramuscular (i.m.) injection of 35 mg/kg ketamine (Pfizer, Madrid, Spain) and 5 mg/kg xylazine (Laboratorios Calier S.A., Barcelona, Spain). The rabbits were placed at the anesthetic plane and were positioned in the left lateral decubitus position. An intravenous line was then placed in the peripheral vein of the ear for anesthetic maintenance with a continuous infusion of 1 μg/kg/h fentanyl and 0.1 mg/kg/h midazolam at an infusion rate of 1 mL/h. The animals were also provided with a fluid therapy of physiological saline solution (B. Braun, Barcelona, Spain) at a rate of 1 to 2 mL/kg/h. If the animal occasionally exited the anesthetic plane during the experiment, an i.m. injection of 35 mg/kg ketamine plus 5 mg/kg xylazine was also administered.

**(iv) Endotracheal intubation.** For endotracheal intubation, the protocol described by Thompson et al. (41) was followed with some modifications. When the rabbit was placed in the anesthetic plane, it was positioned on the preparation table with the head slightly extended over the edge of the table in straight alignment with the spine column. After checking for complete jaw relaxation, by lifting the head up, gauze was used to pull the rabbit’s tongue to the right lower incisors, taking care to avoid trauma from the incisors. Then, while extending the rabbit’s head back and neck forward to maintain an open airway and view the larynx, the previously colonized ETT was slowly introduced to the trachea from the rabbit’s left side until we noticed some resistance (previous confirmation that it was the larynx). To prevent oxygen desaturation, it is important to keep the neck extended as described, to maintain an open airway during intubation. The successful introduction of the tube was confirmed by observing the fogging of the glass/mirror at the proximal of the tube and listening to the airflow. The tube was gradually introduced into the desired position. To secure the ETT, the rabbit was placed on its side and an umbilical tape around the tube was tied.

**(v) Ventilation parameters.** The animals were ventilated using a mechanical ventilator (Serve Ventilator 300, Siemens, Germany) and humidifier (Hamilton-H900; Hamilton Medical, Bonaduz, Switzerland). The mechanical ventilator was used in the neonatal mode and pressure control, with an airway pressure peak of 15 cmH_2_O, a Positive End-Expiratory Pressure (PEEP) of 5, a breathing rate of 44 breaths/min, an inspiration period of 0.35 s, a tidal volume of inspiration of 40 mL, and a concentration of O_2_ of 50%. The inspiratory gases were conditioned through the humidifier heated at 38°C. Throughout the process, the fraction of inspired oxygen (FiO_2_) of the animals was monitored to ensure that it was higher than 95%.

**FIG 7:**
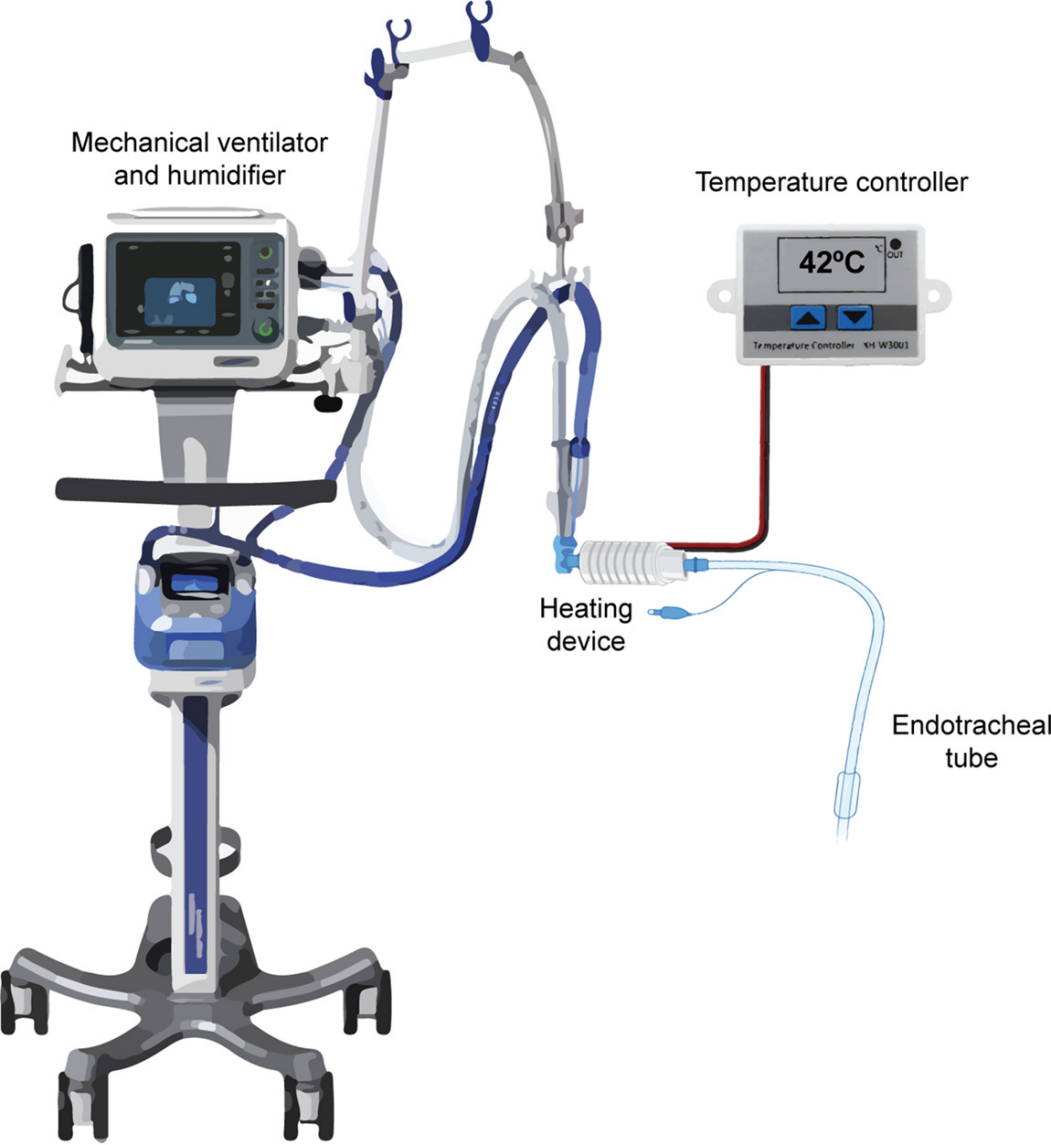
Illustration of the heating device placed between the ETT and the humidifier tubes and connected to a temperature controller. ETT, endotracheal tube.

**(vi) Hyperthermia device.** A heating device (Forbac 100 CE; Sanara) was designed to be attached to a respiratory medical device, such as an ETTs, and the other side was attached to an air ventilation equipment (see Fig. 7). In this manner, the airflow entering the respiratory medical device was homogeneously heated to reach a specific and steady temperature.

The hyperthermia device is formed by an electric resistance in a metallic piece with channels inside that allow airflow. It is recovered by an insulation covering and connected to a temperature controller (Gavalbac 100; Sanara).

This technology has been protected by a utility model (U202032239) and Patent Cooperation Treaty (PCT) application (PCT/EP2021/074309) was performed (42).

**(vii) Application of the hyperthermia.** Forty animals were randomly classified into two groups: control and experimental groups. In the control group (37°C), 20 animals were intubated and ventilated. In the experimental group (42°C), 20 animals were also intubated, ventilated and the heating device was placed between the ETT of the intubated animal and humidifier tubes. The heating device was then plugged into a temperature controller. Three pulses at 42°C for 15 min for each pulse were applied, leaving 15 min without heating the airflow between shots. The temperature inside the ETT was verified during all experiments using a thermometer and thermocouple probe.

All animals were monitored during the experiment, checking that oxygen saturation, body temperature, and heart rate were within the normal range. Unfortunately, it was not possible to keep monitoring the animals immediately after the hyperthermia application and maintain it overnight due to the infrastructures where the experiments were carried out. Furthermore, prior to conducting the experiments, a safety procedure was performed to ensure that hyperthermia and the use of the device would not cause adverse effects in the animals.

**(viii) Evaluation of the hyperthermia efficacy.** Immediately after ventilating the animal for 1 h and 15 min (control animals) or after ventilating the animal and performing three heat pulses (experimental group), the ventilator and its connections were unplugged. The ETT was carefully and aseptically removed from the rabbit, while keeping the animal in the left lateral decubitus position. Once the ETT was completely removed, we checked that the oxygen saturation was still adequate and the animal was able to breathe normally, without making breathing noises. The external part of the ETT was cleaned with 80% alcohol and physiological saline solution using a sterile gauze. The ETT was then locked with TSB and incubated at 37°C for 24 h under stirring (60 rpm) to complete the biofilm growth step. Subsequently, four pieces of 0.5 cm of the distal part of the ETT were cut off and then cut in half. The inner part of each piece was stained using 50 μL of a mixture of the Live/Dead BacLight viability kit for 30 min in the dark and visualized using a Zeiss LSM980 CLSM with excitation wavelengths of 488 and 568 nm. The mixture was prepared with 3 μL of each dye in 1 mL of sterile distilled water.

Three areas of the biofilm on each piece were scanned by using the tiles scanning package at ×40 of the Zeiss LSM980 confocal microscope, and ZEN blue 3.3 software was used. The tiles scanning consists on defining and scanning multiple consecutive tiles of the sample so a larger and more representative area of the sample can be studied. Z-stacks of every single tile were also defined in order to collect the information of all the layers of the formed biofilm. All the multiple scanned tiles were then stitched consecutively to form a single image. Finally, once having the formed image with the stitched tiles, a maximum projection was obtained to have all the z-stacks in a single plane. Simultaneous dual-channel imaging, ZEN Blue 3.3 software, and the ImageJ 1.45s software package were used as previously mentioned in the *in vitro* section. Cell viability is expressed as the percentage mean of the heat pulses versus the control group and compared using a Student *t* test. Statistical analysis was performed using the SPSS. *P* values of ≤0.05 were considered statistically significant.

At 24 h after ventilation (with or without hyperthermia), the animals were sedated by an i.m. injection of 35 mg/kg ketamine plus 5 mg/kg xylazine. When they did not have a corneal reflex and had a loss of station, they were euthanized by an intravenous injection of 200 mg/kg pentobarbital (B. Braun, Barcelona, Spain).
